# Revisiting the Basic Symptom Concept: Toward Translating Risk Symptoms for Psychosis into Neurobiological Targets

**DOI:** 10.3389/fpsyt.2016.00009

**Published:** 2016-01-28

**Authors:** Frauke Schultze-Lutter, Martin Debbané, Anastasia Theodoridou, Stephen J. Wood, Andrea Raballo, Chantal Michel, Stefanie J. Schmidt, Jochen Kindler, Stephan Ruhrmann, Peter J. Uhlhaas

**Affiliations:** ^1^University Hospital of Child and Adolescent Psychiatry and Psychotherapy, University of Bern, Bern, Switzerland; ^2^Developmental Clinical Psychology Research Unit, Faculty of Psychology and Educational Sciences, University of Geneva, Geneva, Switzerland; ^3^Research Department of Clinical, Educational and Health Psychology, University College London, London, UK; ^4^Department of Psychiatry, Psychotherapy and Psychosomatics, University Hospital of Psychiatry, Zurich, Switzerland; ^5^School of Psychology, University of Birmingham, Birmingham, UK; ^6^Norwegian Centre for Mental Disorders Research (NORMENT), Faculty of Medicine, University of Oslo, Oslo, Norway; ^7^Department of Psychiatry and Psychotherapy, University of Cologne, Cologne, Germany; ^8^Institute of Neuroscience and Psychology, University of Glasgow, Glasgow, UK

**Keywords:** basic symptoms, neurobiology, psychosis, clinical high risk, aetiopathology

## Abstract

In its initial formulation, the concept of basic symptoms (BSs) integrated findings on the early symptomatic course of schizophrenia and first *in vivo* evidence of accompanying brain aberrations. It argued that the subtle subclinical disturbances in mental processes described as BSs were the most direct self-experienced expression of the underlying neurobiological aberrations of the disease. Other characteristic symptoms of psychosis (e.g., delusions and hallucinations) were conceptualized as secondary phenomena, resulting from dysfunctional beliefs and suboptimal coping styles with emerging BSs and/or concomitant stressors. While BSs can occur in many mental disorders, in particular affective disorders, a subset of perceptive and cognitive BSs appear to be specific to psychosis and are currently employed in two alternative risk criteria. However, despite their clinical recognition in the early detection of psychosis, neurobiological research on the aetiopathology of psychosis with neuroimaging methods has only just begun to consider the neural correlate of BSs. This perspective paper reviews the emerging evidence of an association between BSs and aberrant brain activation, connectivity patterns, and metabolism, and outlines promising routes for the use of BSs in aetiopathological research on psychosis.

## Introduction

Over the past two decades, preventive research in psychosis has renewed interest in subjective and subclinical psychopathology beyond positive and negative symptoms. One approach to a detailed description of such subtle disturbances, developed since the 1960s, is Huber’s “basic symptoms” (BSs) concept (Figure S1 in Supplementary Material).

### The Concept of Basic Symptoms

Basic symptoms are subtle, subclinical disturbances in stress tolerance, drive, affect, thinking, speech, (body) perception, motor action, and central-vegetative functions that are self-experienced with full insight into their abnormal nature (1, 2). Despite having insight, people find these subjective experiences so new and strange that they remain almost inexplicable, and therefore usually require guided questioning for their assessment. Being different from what is considered to be one’s “normal” mental state, BSs remain predominately private and apparent only to the individual. Thus, rather than BSs themselves, it will be a person’s affective reactions and self-initiated coping strategies in response to their BSs that may be recognized by others. Therefore, BSs differ from (secondary) negative symptoms in their current understanding as dysfunctional mental and behavioral response observable to others (3). Being experienced with full insight, BSs are also distinct from positive symptoms which are experienced by the individual as real, normal thinking, and feeling (2, 4).

Basic symptoms are an integral part of psychosis and appear throughout various stages of the disorder (Figure S1 in Supplementary Material). In combination with selected attenuated psychotic symptoms (APS), a subgroup of BSs was recently conceptualized as “self-disorders” (SDs) and a core schizophrenia vulnerability phenotype [Figures S2 and S3 in Supplementary Material (5–7)].

### Basic Symptoms and Early Neurobiological Research

Huber’s pioneer pneumoencephalographic *in vivo* studies on chronic schizophrenia patients with persistent negative or deficit symptoms led him to initially assume that a deficit syndrome characterized by BSs was caused in most cases by an atrophy of the basal ganglia and inherent small dysplastic lateral ventricles (8). Later on, he put emphasis on the limbic system by conceptualizing BSs as “substrate-close” or “basic,” i.e., the most immediate symptomatic “expression of pathologically cerebral function in the region of the integrative system, which is responsible for the regulation of the cerebral filter and protection processes” [(9) p. 78]. While structural changes would be irreversible and potentially progressive, Huber (9) hypothesized that abnormal rhythms in EEG related to functional structures of the limbic system would only be seen in certain active (particularly early) stages.

### Basic Symptoms and Risk for Psychosis

While studies in the 1980s and 1990s indicated that most BSs are indeed not specific to psychosis and may occur in other, especially non-psychotic affective disorders (4), 14 BSs were specific to the development of first-episode schizophrenia within 9.6 years (10) and employed in two clinical high risk (CHR) criteria (4, 11, 12): Cognitive Disturbances, COGDIS, Cognitive-Perceptive BSs, and COPER (Table 1).

**Table 1:** **CHR criteria according to the BSs concept**.

Cognitive disturbances (COGDIS)
≥2 of the following 9 BSs with at least weekly occurrence (i.e., SPI-A/SPI-CY score of ≥3) within the last 3 months
*• Inability to divide attention* (B1^a^) between a (semi-)automatic and another task that strain different senses, e.g., making a sandwich (visual) while conversing (auditory)
*• Thought interference* (C2) of completely irrelevant, random thought contents
*• Thought blockages* (C3) incl. trailing off mentally and leading to a (temporary) loss of intended thought
*• Disturbance of receptive speech* (C4), i.e., a disturbance in the immediate understanding of verbal stimuli in one’s mother tongue
*• Disturbance of expressive speech* (C5), i.e., in the presence of a clear idea, a disturbance in the immediate access to the adequate word in one’s mother tongue
*• Thought pressure* (D3), i.e., rapid succession of irrelevant, unrelated thoughts
*• Unstable ideas of reference* (D4), experienced with immediate insight
*• Disturbances of abstract thinking* (O3), i.e., initial literal understanding of metaphoric contents or symbols
*• Captivation of attention by details of the visual field* (O7) that are random and irrelevant

**Cognitive-perceptive basic symptoms (COPER)**

≥1 of the following 10 BSs with at least weekly occurrence (i.e., SPI-A/SPI-CY score of ≥3) within the last 3 months and 1st occurrence ≥12 months ago (irrespective of frequency and persistence during this time)
*• Thought interference* (C2)
*• Thought blockages* (C3)
*• Disturbance of receptive speech* (C4)
*• Thought pressure* (D3)
*• Unstable ideas of reference* (D4)
*• Thought perseveration* (O1), i.e., repeated intrusion of irrelevant thought contents
*• Decreased ability to discriminate between ideas/perception, fantasy/true memories* (O2), i.e., unfounded consideration of perceptions or memories as products of current imagination
*• Derealization* (O8), incl. reduction to 2-dimensional vision and increased emotional involvement into the surrounding
*• Visual perception disturbances*, excl. blurred vision and hypersensitivity to light (D5, F2, F3, and O4), i.e., perceptive distortions that are immediately recognized as own misperceptions
*• Acoustic perception disturbances*, excl. hypersensitivity to sounds (F5 and O5), as above

*^a^Item numbers refer to the “Schizophrenia Proneness Instrument, Adult Version (SPI-A)” that gives more extended descriptions of BS and instructions for their assessment (12)*.

A recent meta-analysis (11) revealed pooled conversion rates in COGDIS-defined samples of up to 54.9% within 4 years. Four-year conversion rates of COGDIS samples were significantly higher than those of samples established by ultra-high risk (UHR) criteria (11), mainly by APS. Thus, COGDIS is one of three criteria recommended for CHR assessment by the European Psychiatric Association (11).

### Neurocognition and Basic Symptoms

Neurocognitive deficits are a common feature of schizophrenia and are also reported in CHR samples (13). To date, few studies separately examined BSs samples and reported rather inconsistent findings. Generally, patients exclusively meeting BSs criteria performed intermediate to UHR patients and controls. While neurocognitive deficits and cognitive BSs did not correlate, there is some evidence for an association between exclusive presence of COPER and executive control/verbal memory dysfunction (Table 2) (14). Thus, BSs samples without APS or brief limited intermittent psychotic symptoms (BLIPS) exhibit fewer and less pronounced neurocognitive impairments compared to samples with APS/BLIPS. This might indicate that BSs generally precede neurocognitive impairments.

**Table 2:** **Summary of neurobiological studies of basic symptoms**.

Study	Aims and hypotheses	Sample and assessments	Main results	Discussion and conclusion
**NEUROCOGNITIVE STUDIES**				
Pukrop et al. (15)	Aim: identifying potential biobehavioral risk factors and investigate illness progression within a cross-sectional design Hypothesis: continuous decline of neurocognitive functioning in scope and intensity from COPER/GRFD and APS/BLIPS to FEP and MEP	Sample: COPER/GRFD (*n* = 38), APS/BLIPS (*n* = 90), FEP (*n* = 86), MEP (*n* = 88) Assessments: BSABS, SIPS/SOPS; neurocognitive tests (VBM, CPT-IP, DRT, AVLT, ROFT, WCST, and verbal fluency	COPER/GRFD > APS/BLIPS > FEP > MEP COPER/GRFD had abnormalities in verbal memory (immediate recall) and verbal executive function (verbal fluency)	Results support a neurodevelopmental model of psychosis with further progressive mechanisms and are consistent with a primary involvement of left frontotemporal networks in the prodromal phase	
Simon et al. (16)	Aim: better understanding of cognitive functioning and its course in CHR states of psychosis Hypotheses: (1) patients with BSs show cognitive impairment when compared with normative values and PCo and (2) these deficits are comparable to those observed in patients meeting UHR criteria	Sample: BS (*n* = 24), UHR (*n* = 69), FEP (*n* = 43), PCo (*n* = 49) Assessments: SPI-A, SIPS/SOPS; neurocognitive tests (MWT, LNS, TMT, verbal fluency, WCST, AVLT, and TAP)	BSs patients worse compared with normative data (working memory, verbal fluency), but not compared to PCo BS > UHR	Most pronounced deficits affect executive functions and working memory → frontal lobe dysfunction in CHR groups	
Schultze-Lutter et al. (17)	Aim: possible association between subjective and objective cognitive disturbances and their relation to different CHR states Hypotheses: COPER/GRFD less impaired than APS/BLIPS; Association between subjective and objective cognitive disturbances	Sample: COPER/GRFD (*n* = 33), APS/BLIPS (*n* = 69) Assessments: BSABS/SPI-A, SIPS/SOPS; neurocognitive tests (VBM, CPT-IP, DRT, LNS, SOPT, AVLT, ROFT, TMT, DST, WCST, and verbal fluency)	COPER/GRFD > APS/BLIPS Association between subjectively reduced stress tolerance and processing speed No further correlation between subjective cognitive–perceptive disturbances and performance in neurocognitive tests	Results support earlier findings showing lack of association between neurocognitive deficits and psychopathologic features. Possible additional predictive power of neurocognitive deficits in CHR states
Frommann et al. (14)	Aim: addressing the neurocognitive functions of 2 different CHR groups in comparison to a healthy control group Hypotheses: (1) CHR have generalized neurocognitive deficits compared with HC, (2) Measures of executive function and verbal memory are more impaired than those of other domains in the APS/BLIPS, and (3) Individuals in an COPER/GRFD are intermediate between HC and APS/BLIPS	Sample: COPER/GRFD (*n* = 116), APS/BLIPS (*n* = 89), HC (*n* = 87) Assessments: ERIraos; neurocognitive tests (MWT, CPT-IP, LNS, SOPT, AVLT, TMT, DST, and verbal fluency)	HC > COPER/GRFD > APS/BLIPS In COPER/GRFD executive control was significantly more impaired in comparison to the remaining domains. In the APS/BLIPS the verbal memory domain was more impaired in comparison to the remaining domains	Executive control seems to be compromised in the COPER/GRFD (prior to the onset of positive symptoms), whereas verbal memory dysfunctions appear to evolve during a later prodromal stage		
Simon et al. (18) [follow-up of Simon et al. (16)]	Aim: long-term follow-up of CHR individuals and their cognitive performance. Comparing individuals who later convert to psychosis with those who do not convert to psychosis Hypotheses: BSs individuals are less cognitively impaired than UHR individuals. UHR individuals that remit from an initial UHR status show cognitive impairment that is at intermediate position between BSs and non-remitting or converting UHR individuals	Sample: BS (*n* = 26), UHR_rem_ (*n* = 35), UHR_n-rem_ = 19, FEP (*n* = 48), PCo (*n* = 49) Assessments: SPIA, SIPS/SOPS; neurocognitive tests (MWT, LNS, TMT, verbal fluency, WCST, AVLT, and TAP) At baseline, global cognitive functioning showed an increase of impairment from PCo to FEP (mean sum score of cognitive functioning: PCo > BS > UHR_rem_ > _UHRn-rem_ > FEP)	At baseline BS group was impaired, but less than UHR group (verbal memory, verbal fluency, executive functions)	Even in the absence of psychotic symptoms cognitive functioning, including executive functioning, was impaired in this CHR sample, this calls for strong efforts to address and remediate cognitive impairments as early as possible in CHR patients
Koutsouleris et al. (19)	Aim: can multivariate neurocognitive pattern classification facilitate the diagnostic identification of different CHR states for psychosis and facilitate an individualized prediction of illness transition Hypotheses: (1) the employment of a support vector machine in conjunction with ensemble learning methods facilitates recognition of different CHR states and the prediction of frank psychosis. (2) Potentially complex patterns of cognitive ability derived from the combination of several neuropsychological tests may also facilitate the individualized prediction	Sample: HC (*n* = 30), COPER/GRFD (*n* = 20), APS/BLIPS (*n* = 28) Assessments: BSABS, CAARMS; neurocognitive tests (MWT, LNS, SOPT, DS, AVLT, TMT, DST, and verbal fluency)	COPER/GRFD patients performed worse in spatial working memory (SOPT) and, processing speed (TMT-A) and executive functions (TMT-B) compared to HC The discriminative pattern of HC vs. COPER/GRFD showed high selection probabilities (>90%) in the working memory and verbal learning/memory domain	The binary classification results suggest that a pattern of altered verbal and mnemonic functions may reliably distinguish CHR individuals experiencing predictive basic symptoms from healthy controls
Haug et al. (20)	Aim: explore the relationships between SDs, as measured by the EASE, and neurocognitive test performance in the early phase of schizophrenia Hypothesis: there are some associations between SDs and neurocognitive deficits, and that higher SDs would correlate with poorer neurocognitive performance	Sample: SZ (*n* = 57) Assessments: EASE; neurocognitive tests (DST, LNS, Logical Memory Test of WMS, ROFT, and DKEFS)	EASE total score was significantly associated with verbal memory (high levels of SDs were associated with impaired verbal memory) No association with SDs and working memory, executive function or psychomotor speed	General lack of associations between SDs and neurocognition is that SDs and these specific neurocognitive functions could represent different basic expressions of the illness Neurocognitive test situation is structured with little affective and somatosensory salience. In contrast, the questions asked in the EASE have focus on more subjective experiences in everyday situations where somatosensory and affective processes interact with neurocognition
Nordgaard et al. (21)	Aim: explore potential associations between SDs, neurocognitive performance, rationality and IQ in patients with schizophrenia Hypothesis: there are some associations between SDs and neurocognitive performance	Sample: SZ (*n* = 31) Assessments: EASE; neurocognitive tests (subtests from the Cambridge Neuropsychological Test Automated Battery)	No significant correlation was found between SDs and neurocognitive performance SDs correlate significantly with rationality (tested with syllogism test)	The general lack of associations between SDs and neurocognitive performance suggests that these phenomena represent different aspects of the disorder – i.e., SDs seem to reflect aspects that are essential or specific to schizophrenia, whereas impaired neurocognitive performance does not The association between rationality and SDs could signify, that high levels of SDs make the patient insensitive to detect violations of logic
**NEUROCHEMICAL AND DRUG STUDIES**				
Korver et al. (22)	Aim: investigation of the relationship between cannabis use, UHR symptoms and neuropsychology Hypothesis: Cannabis-using control subjects and UHR subjects show increased symptomatology and reduced neuropsychological functioning compared to non-using subjects	Sample: UHR subjects (*n* = 63, of them 34 cannabis users), HC (*n* = 58, of them = 28 cannabis users) Assessments: SIPS, BSABS-P, CIDI (sections J and L); neurocognitive tests (FTT, CPT, CVLT, National Adult Reading Test, and verbal fluency)	More basic symptoms and UHR symptoms in cannabis-using UHR subjects compared to non-using UHR subjects Cannabis-using control group showed more subclinical UHR, basic symptoms and more dysfunction than non-cannabis control subjects Frequency of cannabis use correlated with severity of several UHR symptoms No significant relationship between frequency of cannabis use and any neuropsychological test.	The association between frequency of cannabis use and UHR symptoms led to the assumption that frequent use of cannabis is related to changes in visual information processing Frequent use of cannabis could represent a risk factor for developing subclinical UHR symptoms and impaired neurocognitive functioning in healthy subjects
Morgan et al. (23)	Aim: (1) Assess the degree of basic symptoms in currently non-psychotic users of 3 classes of drugs, namely cannabis (high-potency cannabis), stimulants (cocain) and dissociative anesthetics (ketamine). (2) Investigate measures that have shown sensitivity to cognitive deficits in prodromal individuals Hypothesis: are BSs and neurocognitive deficits present in individuals dependent on these drugs? Are BSs associated with drug use *per se* or do users of these different pharmacological agents display differing profiles?	Sample: *N* = 130; dependent high-potency cannabis users (*n* = 29), dependent cocaine users (*n* = 22), dependent ketamine users (*n* = 21), recreational drug users (*n* = 28), drug-naïve control (*n* = 30) Assessments: SPI-A; neurocognitive tests (RBMT and STW)	Deficits in working memory were only found in ketamine users and deficits in frontal functioning in ketamine and high-potency cannabis users. Long-term memory was impaired in all drug users The symptom profile associated with chronic ketamine use was similar to individuals with basic symptoms who subsequently make a transition to psychosis	Ketamine, high-potency cannabis and cocaine users showed basic symptoms, whereas ketamine users exhibited highest levels of basic symptoms The existence of basic symptom-like phenomena is a potential mechanism by which heavy drug use triggers acute psychosis in vulnerable individuals
Koethe et al. (24)	Aim: to evaluate whether changes in the endocannabinoid system [i.e., Anandamide in cerebrospinal fluid (CSF)] are detectable in initial prodromal states of psychosis Hypothesis: elevation of Anandamide in CSF is apparent in early stages of psychosis	Sample: HC (*n* = 81), UHR (*n* = 27) Assessments: SPI-A, PANSS, SOPS	Cerebrospinal Anandamide levels in patients were significantly elevated. Patients with lower levels showed a higher risk for transiting to psychosis earlier	The up-regulation of Anandamide in the initial prodromal course suggests a protective role of the endocannabinoid system in early schizophrenia
Huang et al. (25)	Aim: to evaluate whether CSF alterations of glucose, lactate, VGF and transthyretin, that have been found in SZ, are already detectable in UHR Hypothesis: none stated	Sample: FEP, drug naive (*n* = 54); UHR (*n* = 24); HC (*n* = 70) Assessments: SPI-A, PANSS, SIPS/SOPS	~1/3 of UHR patients displayed proteomic/metabolic profiles characteristic of FEP, drug naive, i.e., changes in levels of glucose, lactate, VGF-derived peptide (VGF23-62) and transthyretin	Schizophrenia-related biochemical disease processes can be traced in CSF of prodromal patients
**ELECTROPHYSIOLOGICAL STUDIES**				
Wölwer et al. (26)	Aim: to investigate impairments of facial affect recognition and its neurophysiological correlates in two different CHR states Hypothesis: CHR individuals show poorer affect recognition performance and abnormalities in ERP components as correlates of impaired encoding of facial features and affect decoding processes	Sample: HC (*n* = 32), COPER/GRFD (*n* = 16), APS/BLIPS (*n* = 21) Assessments: ERIraos Pictures of Facial Affect (6 basic emotions) EEG: event-related potentials (ERP: P100, N170, N250)	Facial affect recognition in CHR < HC, no significant difference between CHR groups Amplitudes of all three ERPs in CHR < HC (CHR groups were collapsed for ERP analysis)	(1) The ability to discriminate emotional expressions in faces is impaired in the CHR state (COPER/GRFD as well as APS/BLIPS), demonstrating an impairment of social cognition already before the first psychotic episode (2) Reduced N100 may be due to an impairment of fundamental visual processes, N170 may reflect dysfunctions in visual processing of facial structures. Diminished N250 amplitudes may indicate difficulties to associate the structural representation of the face with semantic and contextual information
Frommann et al. (27)	Aim: to determine whether individuals in two different CHR states show P300 amplitude reductions and altered topography Hypothesis: CHR individuals in both states show left temporoparietal amplitude reduction compared to controls	Sample: HC (*n* = 40), COPER/GRFD (*n* = 50), APS/BLIPS (*n* = 50) Assessments: ERIraos EEG: ERP P300, oddball paradigm	Hit rate: APS/BLIPS = HC, COPER/GRFD = HC* P300 latency: APS/BLIPS = HC, COPER/GRFD = HC* *COPER/GRFD vs. APS/BLIPS not reported P300 amplitude: sagittal midline (SM) and left hemisphere electrodes: APS/BLIPS < HC, COPER/GRFD = HC, APS/BLIPS = COPER/GRFD Sagittal midline: BLIPS positive < BLIPS negative Left temporoparietal electrode: COPER/GRFD < HC	P300 activity in the COPER/GRFD differed only at left temporoparietal position from HC, whereas in the APS/BLIPS, markedly amplitude reductions were observed, pronounced over the left hemisphere Findings may indicate a disturbance of neural generators in the left superior temporal lobe occurring early in the disease process. Temporoparietal P3 reductions may indicate vulnerability to psychosis. Sagittal midline P3 amplitudes may reflect changes underlying the development of psychotic symptoms
Arnfred et al. (28)	Aim: explore potential associations between SDs and abnormalities of early contralateral proprioceptive evoked oscillatory brain activity Hypothesis: none stated	Sample: SZ (*n* = 12) Assessments: EASE EEG: proprioceptive-evoked potentials	Higher EASE scores (i.e., increased SDs) were associated with lower peak parietal gamma frequencies and higher peak beta amplitudes over frontal and parietal electrodes in the left hemisphere following right-hand proprioceptive stimulation	SDs may be associated with dysfunction of early phases of somatosensory processing
Sestito et al. (29)	Aim: to investigate the relation between SDs and subtle, schizophrenia-specific impairments of emotional resonance that are supposed to reflect abnormalities in the mirror neurons mechanism. To test whether electromyographic response to emotional stimuli (i.e., a proxy for subtle changes in facial mimicry and related motor resonance mechanisms) would predict the occurrence of anomalous subjective experiences (i.e., SDs) Hypothesis: none stated	Sample: SZ spectrum (*n* = 18) Assessments: BSABS EMG: multimodal paradigm, recording facial electromyographic activity of muscles in response to positive and negative emotional stimuli	SZ spectrum patients showed an imbalance in emotional motor resonance with a selective bias toward negative stimuli, as well as a multisensory integration impairment. Multiple regression analysis showed that electromyographic facial reactions in response to negative stimuli presented in auditory modality specifically and strongly correlated with SDs subscore	The study confirms the potential of SDs as target phenotype for neurobiological research and encourages research into disturbed motor/emotional resonance as possible body-level correlate of disturbed subjective experiences in SZ spectrum
Sestito et al. (30)	Aim: to explore whether a low or high emotional motor resonance occurring in SZ spectrum relates to clinical features and BSs Hypothesis: none stated	Sample: SZ spectrum (*n* = 19) Assessments: BSABS EMG: multimodal paradigm, recording facial electromyographic activity of muscles in response to positive and negative emotional stimuli	SZ spectrum patients more resonating with negative emotional stimuli (i.e., externalizers) had significantly higher scores in BSABS Cluster 3 (vulnerability) and more psychotic episodes than internalizers patients. SzSp patients more resonating with positive emotional stimuli (i.e., externalizers) scored higher in BSABS Cluster 5 (interpersonal irritation) than internalizers	Abnormal subjective experiences are related to low-level emotional motor mechanisms disruption, indexed by electromyographic facial reactions
Van Tricht et al. (31)	Aim: quantitative EEG spectral power and alpha peak frequencies (APF) were determined in CHR subjects Hypothesis: none stated	Sample: CHR (*n* = 113), HC (*n* = 25) Assessments: SPI-A, SIPS/SOPS EEG: Ag/AgCl electrodes were applied according to the international 10–20 system; individual APF were assessed	Compared to CHR without transition HC, CHR with transition showed higher theta and delta on frontal and central scalp locations and lower occipital-parietal APF. Furthermore, in CHR without transition, upper parietal alpha was lower compared to HC. A model for prediction of psychosis included frontal theta and delta as well as the APF as predictors of 18-month conversion rates	Theta and delta ranges and APF can contribute to the short-term prediction of a first psychotic episode
Andreou et al. (32)	Aim: investigate EEG resting-state connectivity in CHR compared to SZ spectrum and HC, and its association with cognitive deficits Hypothesis: none stated	Sample: CHR (*n* = 28), SZ spectrum (*n* = 16), HC (*n* = 23) Assessments: SPI-A, SIPS/SOPS; Neurocognitive tests (VLMT, WMS, TMT, LNS, DS, DST, verbal fluency) EEG: 64-channel resting state EEG recordings (eyes closed).	SZ displayed increased theta-band resting-state multivariate interaction measure connectivity across midline, sensorimotor, orbitofrontal regions and the left temporoparietal junction. CHR displayed intermediate theta-band connectivity patterns that did not differ from either SZ or HC: mean theta-band connectivity within the above network partially mediated verbal memory deficits in SZ and CHR	Aberrant theta-band connectivity may represent a trait characteristic of schizophrenia associated with neurocognitive deficits
Ramyead et al. (33)	Aims: to assess whether abnormalities in current source density (CSD) and lagged phase synchronization of oscillations across distributed regions of the brain already occur in patients with CHR state for psychosis Hypotheses: (1) CHR with transition would demonstrate abnormal CSD in both the high gamma and beta frequency bands when compared with CHR without transition and HC. (2) The lagged phase synchronization of beta, the long-range modulator, would be more decreased in CHR with transition compared to CHR without transition and HC as a function of increasing Euclidian distance	Sample: CHR (*n* = 63), HC (*n* = 29) Assessments: BSIP; neurocognitive tests (TAP) EEG: resting-state EEG	CHR with transition showed higher gamma activity in the medial prefrontal cortex compared to HC, which was associated with abstract reasoning abilities in CHR with transition. Furthermore, in CHR with transition lagged phase synchronization of beta oscillations decreased more over Euclidian distance compared to CHR without transition and HC. Finally, this steep spatial decrease of phase synchronicity was most pronounced in CHR with transition patients with high positive and negative symptoms scores	Patients who will later make the transition to psychosis are characterized by impairments in localized and synchronized neural oscillations providing new insights into the pathophysiological mechanisms of schizophrenic psychoses and may be used to improve the prediction of psychosis
**IMAGING STUDIES: STRUCTURAL**				
Hurlemann et al. (34)	Aims: to which extent interrelated structural–functional deficits of the hippocampus reflect a vulnerability to schizophrenia? Hypothesis: hippocampal volume reduction should be paralleled by a progressive worsening of verbal learning and memory	Sample: COPER/GRFD (*n* = 20), APS/BLIPS (*n* = 16), HC (*n* = 30) Assessments: ERIraos; neurocognitive tests (MWT and AVLT)	Hippocampal volume decrease in COPER/GRFD of 7.7% In APS/BLIPS but not in COPER/GRFD, a 9.2% deficit in AVLT (delayed recall) was correlated with reduced MRI hippocampal volumes	Progressive and interrelated structural–functional pathology of the hippocampus could be an index of increased risk for schizophrenia
Koutsouleris et al. (35)	Aims: (1) to investigate structural brain differences between participants with COPER/GRFD or APS/BLIPS. (2) To examine associations between structural differences and later disease conversion Hypothesis: no hypothesis stated	Sample: COPER/GRFD (*n* = 20), APS/BLIPS (*n* = 26), HC (*n* = 75); 4-year follow-up (total *n* = 33; 13 for COPER/GRFD and 20 for APS/BLIPS), 15 transitioned to psychosis [COPER/GRFD (*n* = 1), APS/BLIPS (*n* = 14)]	Gray matter reductions (controls > COPER/GRFD) in fusiform, superior, middle and inferior temporal gyri, as well as amygdala and hippocampus, bilaterally. For COPER/GRFD > APS/BLIPS, differences in frontal clusters in left subgenual anterior cingulate cortex as well as in the ventromedial prefrontal cortex and dorsomedial prefrontal cortex, bilaterally	BSs are associated with medial and lateral temporal lobe abnormalities, as well as subtle perisylvian, prefrontal, parietal, thalamic and cerebellar anomalies; APS/BLIPS mark are characterized by more pronounced structural anomalies within these regions
Koutsouleris et al. (36)	Aims: to investigate the ability of support vector machines (SVMs) to detect different CHR states by performing a classification of HC vs. individuals with CHR (grouped into COPER/GRFD and APS/BLIPS) and to further evaluate SVMs’ performance in predicting transition in the CHR converting to clinical disorders Hypothesis: None stated	Sample: COPER/GRFD (*n* = 20), APS/BLIPS (*n* = 25), HC (*n* = 25); follow-up 13 for COPER/GRFD, and 20 for APS/BLIPS, 15 transitioned to psychosis [COPER GRFD (*n* = 1), APS/BLIPS (*n* = 14)]	Multivariate neuroanatomical pattern classification can accurately discriminate between COPER/GRFD, APS/BLIPS, and HC. COPER/GRFD patterns appear be distinguishable from HC on the basis of gray matter patterns of both augmentations and reductions in temporal lobe regions. They differ from APS/BLIPS on the basis of gray matter patterns around the cingulate cortex and the perisylvian fissure	COPER/GRFD without other CHR criteria, appear to be distinguishable from both HC and APS/BLIPS subgroups; however, the pattern linked to conversion is not as clear in COPER/GRFD as it is in APS/BLIPS. This is partly due to the fact that a very low proportion of COPER/GRFD patients converted to psychosis (1 on 20) in this study
Koutsouleris et al. (37)	Aims: to test the “accelerated aging” hypothesis across different psychiatric disorders, using brain age gap estimations; to employ multivariate pattern analysis (MPVA) to estimate classifiers’ ability to distinguish between different pathologies Hypothesis: no hypothesis stated	Sample: COPER/GRFD (*n* = 21), APS/BLIPS (*n* = 68), major depression (*n* = 104), borderline personality disorder (*n* = 57), SZ (*n* = 141), HC (*n* = 437)	Results yield negative brainage effects in the COPER/GRFD group	It appears that the COPER/GRFD group showed “decelerated brain aging”; the authors suggest this effect could be due to a maturational delay mechanism, or a compensatory neural mechanism at the early stage of the disease
Tepest et al. (38)	Aims: to investigate interhemispheric connectivity, using measures of the corpus callosum (CC); to investigate corticocortical connectivity, using a gyrification index (GI) measure Hypotheses: changes in both measures reflecting impairments in long distance as well as in short distance connectivity, in comparison with HC subjects	Sample: CHR (*n* = 21), SZ (*n* = 21), HC (*n* = 21)	GI frontal region SZ > HC (+20%) SZ > CHR (+9%) CHR > HC (+10%) GI parietal region SZ > HC (+15%) SZ > CHR (+8%) CHR > HC (+7%)	Results suggest an impairment in short-range corticocortical connectivity, whereas no impaired long-range connectivity no difference in CC measurements
**IMAGING STUDIES: FUNCTIONAl**				
Ebisch et al. (39)	Aims: do FEP patients show functional activation abnormalities during social perception of other individuals’ affective tactile stimulation? Hypothesis: none stated	Sample: FEP (*n* = 24), HC (*n* = 22) Assessments: SPI-A, PANSS fMRI: social perception task using videos depicting animate/inanimate individuals using tactile stimulation	Ventral premotor cortex activation negatively correlates with SPI-A basic symptom scores (0–150)	Results likely reflect poor multisensory integration in the vPMC (visual, tactile, proprioceptive self-experiences)
Ebisch et al. (40)	Aims: investigate connectivity underlying the link between aberrant self-experience and social cognition in FEP Hypothesis: none stated	Sample: FEP (*n* = 24), HC (*n* = 22) Assessments: SPI-A, PANSS fMRI: social perception task using videos depicting animate/inanimate individuals using tactile stimulation	Connectivity between ventral premotor cortex and posterior cingulate cortex correlates with SPI-A basic symptom scores (0–150)	Increased functional coupling between antagonistic functional networks may alter functional segregation, thereby disturbing the relationship between the intrinsic (self-referential) and extrinsic (interacting) self
Wotruba et al. (41)	Aims: to examine whether salience network (SN) disturbances can be evidenced in CHR. Furthermore, to explore if within and between intrinsic functional connectivity in the SN, default mode network (DMN) and task-positive network (TPN) are associated to symptoms related to reality distortions and cognitive processing in CHR subjects Hypothesis: clinical symptoms and disturbances of cognition seen in CHR subjects are reflected by an aberrant spatial extent in DMN, TPN, and SN, accompanied by a loss of anticorrelation between those 3 networks	Sample: BS (*n* = 28), UHR (*n* = 19), HC (*n* = 29) fMRI: resting-state paradigm	mPFC–rDLPFC connectivity, as well as rAI-PCC connectivity increased in BSs and UHR vs. HC (anticorrelated for controls). Significant anticorrelation between the task-positive network (bilateral fronto-parietal) and DMN for HC, but not CHR groups	Absence of typical anticorrelated patterns may relate to irregularities in discrimination between external and internal sources of information, thereby potentially leading to risk symptoms. Note however that no significant differences were found between BSs risk and UHR (UHR seems to show trend-like increased connectivity in rAI-PCC)
Wotruba et al. (42)	Aims: explore functional brain correlates during both anticipation and receipt of rewards and to evaluate their association with symptoms in unmedicated persons at risk for psychosis Hypotheses: (1) positive symptoms are associated with activation of the ventral striatum (VS) and the anterior insula during reward anticipation, (2) negative symptoms are associated with reduced VS activation during reward anticipation, and (3) depressive symptoms are associated with reduced VS and mOFC activation during processing of rewarding outcomes	Sample: CHR (*n* = 21) meeting UHR + BS criteria, HC (*n* = 24) fMRI: monetary incentive delay task to probe neural responses for reward anticipation and receipt	During reward anticipation, increased in CHR: PCC, SFG, medial frontal gyrus. No correlations with BSs, but with SIPS positive in ventral striatum and rAI (positive correlation) No group differences for receipt of reward contrast, correlations with psychopathology: left ventral striatum with negative symptoms (negative correlation)	Evidence for dysregulation of reward processing in risk period, with frontal compensation. Higher striatal activation might be linked to “increased salience” hypothesis in early stages
Ferri et al. (43)	Aim: to examine embodied simulation as driven by mirror neuron in schizophrenia Hypothesis: none stated	Sample: SZ (*n* = 22), HC (*n* = 22) fMRI: goal-related actions in either a neutral or emotional context	Lower activation of the left inferior parietal lobule when observing neutral action correlated with increased basic symptoms score	Emotional cues might allow SZ patients to recover mirror neuron-driven embodied simulation at least in part. However, their understanding of the emotional components of others’ actions will likely remain deficient

*SPI-A, Schizophrenia Proneness Instrument, Adult version; SPI-CY, Schizophrenia Proneness Instrument, Child and Youth version; BSABS, Bonn Scale for the Assessment of Basic Symptoms; EASE, Examination of Anomalous Self-Experience; ERIraos, Early Recognition Inventory/Interview for the Retrospective Assessment of the Onset of Schizophrenia; SIPS/SOPS, Structured Interview for Prodromal Syndromes; CAARMS, Comprehensive Assessment of At Risk Mental States; CHR, clinical high risk; BSs, basic symptoms; SDs, self-disturbances; UHR, ultra-high risk; FEP, first-episode psychosis; MEP, multiple episode psychosis; SZ, schizophrenia; PCo, patient controls; HC, healthy controls*.

*Neurocognitive tests: MWT, Mehrfach–Wortschatz-Test; CPT-IP, Continuous Performance Test-Identical Pairs version; TAP, Testbatterie zur Aufmerksamkeitsprüfung; DRT, Delayed Response Task; LNS, Letter-Number Span; DS, Digit Span Test; SOPT, Subject Ordered Pointing Task; AVLT, Rey Auditory Verbal Learning Test; ROFT, Rey–Osterrieth Complex Figure Test; WMS, Wechsler Memory Scale; TMT, Trail-Making Tests; DST, Digit Symbol Test; WCST, Wisconsin Card Sorting Test; RBMT, Rivermead Behavioural Memory Test; STW, Spot The Word Test; FTT, Finger-Tapping Test; CVLT, California Verbal Learning Test*.

*Neuroimaging: vPMC, ventral premotor cortex; mPFC, medial prefrontal cortex; rDLPFC, right dorsolateral prefrontal cortex; PCC, posterior cingulate cortex; rAI, right anterior insula; SFG, superior frontal gyrus; mOFC, medial orbitofrontal cortex*.

## Basic Symptoms and Current Neurobiological Research in Psychosis

### Neurochemistry and Basic Symptoms

Neurochemical findings suggest a role for dopaminergic, glutamatergic, serotonergic, and GABAergic systems in schizophrenia (44, 45). *In vivo* research on CHR states, defined by either UHR or BSs criteria, has focused mainly on dopamine (46, 47), glutamate (48, 49), and GABA (50, 51). Currently, the strongest evidence comes from Positron Emission Tomography studies indicating an increase of presynaptic striatal dopamine synthesis in APS patients compared with controls (46, 47). In addition, an increased dopamine synthesis capacity was also reported for individuals at genetic high risk for schizophrenia (52). Dopamine studies in BSs samples however are still missing.

These neurochemical studies have been complemented by pharmacological models of psychosis, e.g., the ketamine and endocannabinoid models (22, 23). Ketamine is an NMDA receptor antagonist, whereas cannabis or delta-9 tetrahydrocannabinol (THC) is an agonist on the cannabinoid receptor CB1. While commonly the effect of substances has to be ruled out to rate subjective experiences as BSs, recent studies ignoring this rule demonstrated an association of cannabis and ketamine use with cognitive and perceptive BSs (22, 23). Cannabis-using CHR patients had more BSs than non-using patients (22), while non-CHR cannabis users had significantly more positive, disorganization, general symptoms and BSs and also more neurocognitive deficits than non-users (23). Furthermore, the profiles of BSs and neurocognitive deficits of high-potency cannabis and ketamine users resembled COPER patients who subsequently converted to psychosis more closely than the profiles of users of other substances (23). Additional support of an association between the endocannabinoid system and BSs comes from one study investigating anandamide a bioactive lipid binding to cannabinoid receptors, in the cerebrospinal fluid (CSF) of CHR states (24). CHR individuals with higher anandamide levels showed a lower risk for transitioning to psychosis. Finally, one study of the metabolic profile in CSF (25) reported increased levels of glucose and VGF peptide (a polypeptide expressed by neurons and neuroendocrine tissues), and decreased levels of lactate and transthyretin protein in CHR patients (Table 2).

### Electrophysiology and Basic Symptoms

EEG and magnetoencephalographic (MEG) recordings permit the non-invasive assessment of electric currents of large populations of neurons, thus providing an estimate of both spontaneous and task-induced activity with millisecond resolution.

In a study of event-related potentials (ERP: P100, N170, and N250) using a facial recognition paradigm (26), emotion recognition was reduced in CHR groups and accompanied by a decrease in ERP amplitudes. As emotion recognition is already completed within 100 msec (53), these findings may reflect more complex perceptual processes. Further research on their relation to visual BSs may be promising. Moreover, reduced P300 amplitudes during an auditory oddball paradigm were found in a CHR sample (27). The COPER group showed a significant lower amplitude at a left temporoparietal site, whereas the APS/BLIPS group elicited smaller amplitudes at midline and left hemispheric electrode sites. These findings suggest potential differences in ERPs between BSs-defined and APS-defined CHR patients that might be related to different states of disturbed information processing.

In addition, Arnfred et al. (28) examined correlations between proprioceptively evoked event-related responses and changes in SDs in a small sample of schizophrenia patients. EEG data were examined for the spectral power of evoked-activity at beta/gamma-band frequencies (13–80 Hz) in response to a sudden change in muscle contraction. Increased total scores as well as increased ratings in the subscales “cognition and stream of consciousness,” “self-awareness and presence” and “bodily experiences” correlated significantly with lower gamma-band activity over parietal electrodes and higher peak frequencies in beta-activity (Table 2). Beside task-related activity, resting-state neural oscillations have also been recently investigated in CHR samples defined by both APS and BSs (31, 32), indicating increased delta/theta-band activity with reduced alpha-band power (31) and reduced theta-band activity which correlated with neurocognitive impairments (32), respectively. Moreover, there is emerging evidence that spontaneous gamma-band activity may differentiate CHR patients from controls (33).

These findings provide preliminary evidence for a potential link between BSs and abnormalities in EEG parameters in patients with schizophrenia and CHR groups. However, it is essential for these findings to be replicated and expanded in large samples. Overall, data on aberrant neural oscillations and ERP-parameters are consistent with data from APS-only (54) and schizophrenia samples (55), suggesting a continuum between psychosis-risk and progression to ScZ. Thus, neural oscillations and their synchronization could constitute a candidate mechanism for BSs. During normal brain functioning, rhythmic activity, especially at gamma-frequency ranges, are important for ensuring effective communication between and within neuronal assembles and correlate with a range of cognitive processes, including attention, perception, and working memory (55). Moreover, 30–80 Hz activity is generated by the interplay between GABAergic-interneurons and excitatory drive mediated through NMDA/AMPA-receptors (56, 57). These cellular mechanisms have been shown to be disrupted in schizophrenia (58).

### Functional and Structural Imaging and Basic Symptoms

To date, a handful of magnetic resonance imaging (MRI) studies have included the assessment of BSs by specialized instruments (Table 2). Their findings were similar to those reported for UHR and schizophrenia patients (59–61).

#### Structural Studies

Five studies have investigated structural characteristics in relation to BSs. These studies distinguished “early risk” for psychosis, characterized by either COPER or the UHR genetic risk criterion (GRFD) in the absence of symptomatic UHR states, from “late risk,” which encompasses individuals meeting APS or BLIPS criteria, irrespective of the presence of BSs (34–37). Hurlemann et al. (34) reported bilaterally reduced hippocampi in COPER/GRFD and in APS/BLIPS subjects, correlating in the latter group with delayed recall in a verbal memory test. Koutsouleris et al. (35) employed voxel-based morphometry analyses to examine morphological differences between early- and late-risk samples. In comparison to controls, the COPER/GRFD group presented gray matter reductions involving the fusiform, superior, middle, and inferior temporal gyri, as well as amygdala and hippocampus, bilaterally. While they were associated with medial and lateral temporal lobe abnormalities, as well as subtle perisylvian, prefrontal, parietal, thalamic, and cerebellar anomalies, these alterations were continuous with late-risk participants (35). In a parallel study using multivariate neuroanatomical pattern classification, morphological patterns of COPER/GRFD were distinguishable from controls on the basis of gray matter patterns of both augmentations and reductions in temporal lobe regions; they differed from late risk on the basis of gray matter patterns covering the anteroposterior cingulate cortex and the perisylvian fissure (36). Using brain age gap estimations, the same group of participants reporting COPER/GRFD presented a “decelerated brain aging,” suggesting differential maturational dynamics at different stages of risk. Such deceleration could be due to a maturational delay mechanism, or a compensatory neural mechanism at the early stage of the disease (37). A further study indicated increased gyrification in frontal and parietal regions in BSs individuals (identified using COGDIS) compared to controls, implicating impaired short-range corticocortical connectivity (38).

#### Functional Imaging Studies

To date, only five fMRI studies have examined BSs (39–43). Studies examining cerebral activation in patients with schizophrenia consistently reported significant associations between BSs and localized decreased activations in the ventral premotor cortex (40) and in the left inferior parietal lobule during passive viewing of neutral actions (43). Notably, increased connectivity between ventral premotor cortex and posterior cingulate cortex was associated with the severity of BSs in first-episode schizophrenia patients (39). This is consistent with another study examining the relationship between task positive and default-mode connectivity in CHR subjects, which reported a lack of anticorrelation between task positive and task negative networks (41).

The preliminary nature of these studies does not enable any definite conclusion as to the links between altered functional connectivity and BSs. Differences in methodology as well as heterogeneity of samples, which included both CHR and frankly psychotic patients, limit the interpretation of the available results and likely contributed to their inconsistencies (62). Furthermore, the variety of BSs involved, which touch upon motivation, cognitive and perceptual domains are likely to involve a variety cortical regions and networks. Overall, these pioneering studies suggest atypical patterns of neural activation in relation to BSs in terms of both reduced activity in discrete regions associated to self-other boundary distinction and atypical cross-talk between networks, which is similar to what is observed in UHR populations (59).

## Discussion and Perspectives

### Current Evidence

Although, in their original formulation, BSs were thought to reflect core abnormalities in brain functioning, investigations have only recently begun to look for their neurobiological origins, especially in CHR groups. Given the importance of BSs criteria in CHR research (11), further studies are needed to provide more detailed insights into the underlying neurobiological correlates that give rise to self-experienced disturbances in perception and cognition. Such candidate mechanisms could be of crucial importance for understanding the etiology of psychosis-risk as well as provide potential biomarkers for early detection and diagnosis. Moreover, such insights could point toward useful targets for novel pharmacological and psychological interventions that might ultimately reduce conversion rates.

### Future Studies into the Neurobiology of Basic Symptoms

Current studies provide only preliminary evidence for neurobiological mechanisms underlying BSs. Available data highlights that diverse anatomical, pharmacological and functional correlates may be involved in the manifestation of BSs in psychotic and CHR individuals. These include structural alterations, changes in ERPs and neural oscillations, neurotransmitter systems as well as evidence for changes in large-scale networks as assessed with fMRI. For more detailed and possibly mechanistic insights into neurobiology of BSs, different strategies need to be employed.

First, BSs in their original formulation are a heterogeneous set of symptoms comprising disturbances in perception, affect, drive, and cognition. Dimensional analyses of BSs indicated six BSs dimensions in adult psychosis patients that appear stable across various states of the illness [i.e., from the prodromal phase via the first episode to chronic states/relapse (4, 63)]. However, these six dimensions could not be replicated in a sample of early-onset schizophrenia patients, where four slightly different dimensions emerged [Figures S4–S6 and Table S1 in Supplementary Material (4)]. Thus, while past studies have either considered BSs in total (e.g., SPI-A sum score) or only considered COPER or COGDIS, BSs dimensions or even more differentiated BSs cluster (e.g., distinguishing even further between cognitive, speech, visual, and acoustic disturbances) might offer a more appropriate route to detecting neurobiological mechanisms underlying (if related to specific BSs) or further promoting (if related to unspecific BSs) development of psychosis. Furthermore, as recently indicated for single attenuated and manifest psychotic “Ich-Störungen (64), even investigating the neurobiology of single BSs may provide some significant insight. For example, when considering subjective disturbances of receptive and expressive speech as subtle, functional and only transient variants of receptive and amnesic aphasia, respectively (65), on a phenomenological level, it seems promising to investigate the role of brain regions which are highly correlated with theses neurologic syndromes also in patients exhibiting these BS.

Further insights into the origins of BSs may be derived from considering their developmental pattern and relationship to brain development. Psychoses, in particular of the schizophrenia-spectrum, are considered a fundamentally neurodevelopmental disorder involving two critical time windows [early (perinatal) brain development and adolescence] that together produce the symptomatic manifestations of the disorder. In this framework, early developmental insults may lead to dysfunction of specific neural networks that would account for early and (in some cases) trait-like signs and symptoms, which may have little or no clinical significance. The development of a CHR state (e.g., by additional occurrence or an increase in frequency of COGDIS symptoms) might index an ongoing imbalance of excessive synaptogenesis/pruning in critical networks, and ultimately the emergence of diagnostically relevant psychotic symptoms. This is supported by a recent analysis of two general population samples spanning the age of 8–40 years (66). Age seemed to affect the 14 perceptive and cognitive BSs included in COPER and COGDIS differently, indicating an age threshold of perceptive BS in late adolescents, around age 18, and of cognitive BSs in young adulthood, in the early twenties – with higher prevalence but reduced association to functional deficits and presence of mental disorder in the below-threshold groups. Thereby, differential age effects seem to follow normal back-to-front brain maturation processes, during which BSs might occur as temporary, in most cases infrequently occurring non-pathological disturbances. Their persistence or onset after conclusion of main brain maturation processes, however, might signify aberrant brain processes. Furthermore, an alternative or complementary explanation of this age-related pattern might be given against the background of decreasing brain plasticity after myelination and pruning processes reach maturity in the early 20s (67): BSs developing in childhood and adolescence, or rather their underlying neurobiological aberrations, might be much better compensated for by the still developing brain and thus, despite the larger number of affected youths, might only be reported by them as infrequent, momentary phenomena. These assumptions might be examined in future studies (i) by cross-sectionally comparing brain development in adolescents reporting BSs with and without clinical significance, (ii) by comparing subjects with an onset of BSs before and after age 18, and (iii) by comparing non-converters and converters to psychosis with regard to their onset and course of BSs.

In addition, if BSs reflect brain processes related to psychosis, then manifestation of BSs should closely be linked to genetic risk. Indeed, studies on first-degree relatives (7, 68–70) and schizotypal samples (71–73) indicated increased rates of BSs compared to HCs and non-schizophrenia-spectrum patients, respectively. In first-degree relatives, this involved more specific cognitive, perceptive and stress-tolerance-related BSs in particular (68). A recent genome-wide association study (74) identified 108 genetic variants associated with biological pathways central to the pathophysiology of schizophrenia. Thus, another route of future studies could be to explore links between certain BSs and possibly related risk-variants, e.g., between certain cognitive BSs and SNPs involved in cognition related neurotransmission.

Additional insights into the neurobiological basis of BSs might also be gained by using pharmacological perturbations in healthy samples. Preliminary evidence indicates that ketamine as well as THC may be associated with the expression of BSs (22, 23). Further studies in healthy populations using a range of pharmacological models which target specific cannabinoid, glutamatergic, dopaminergic, and GABAergic systems could provide important information on the contribution of dysfunctions in neurotransmitter systems and manifestation of BSs.

Finally, studying CHR patients at different stages may reveal the neurobiological correlates of BSs and evolution of schizophrenia. Theoretical and empirical evidence suggests that BSs may constitute the earliest signposts, preceding the development of UHR and psychotic symptoms. Recent studies point toward anatomical and electrophysiological differences between BSs and UHR samples; but these findings require replication and extension with functional imaging approaches. Longitudinal investigations of individuals identified on the basis of BSs alone could provide crucial information on their neurobiological correlates and potential progressive pathophysiological processes that might lead eventually to psychosis.

## Conclusion

In conclusion, despite the fact that BSs were in their original (and pioneering) formulation considered to be a direct manifestation of brain abnormalities in patients with schizophrenia, the nature of such abnormalities appear subtle and heterogeneous, requiring sophisticated methods of analyses to be detected. Our review suggests that the CHR paradigm may constitute a fruitful paradigm to investigate the relationship between phenomenologically grounded perceptual and cognitive alterations and underlying abnormalities in the functionality of anatomical and functional brain networks. These insights may not only be useful for an improved general understanding of BSs but may ultimately give critical insights into the development of psychosis, which could be crucial for early diagnosis and intervention. Furthermore, as BSs are not only present in the initial prodromal phase, but throughout the course of the illness, they may also enable new insights into the neurobiological determinants of unfavorable outcomes including functional deterioration.

## Author Contributions

FS-L and PU wrote the outline of the article. All authors managed the literature searches to draft their respective chapters. FS-L drafted the introduction and parts 1 and 3 of the Supplementary Material; CM drafted the chapter on Neurocognition and BSs; AT and JK drafted the chapter on Neurochemistry and BSs; PU drafted the chapter on Electrophysiology and BSs and the discussion; MD drafted the chapter on Neuroimaging and BSs; AR drafted part 2 of the Supplementary Material. All authors contributed to and have approved the final manuscript.

## Conflict of Interest Statement

Stephan Ruhrmann served as a consultant for Boehringer Ingelheim, received Speaker’s honoraria from Otsuka and Johnson & Johnson, and travel support from Servier. All other authors declare that the research was conducted in the absence of any commercial or financial relationships that could be construed as a potential conflict of interest.
