# Supplementary material for: Revisiting the Basic Symptom Concept: Toward Translating Risk Symptoms for Psychosis into Neurobiological Targets
Source: Front Psychiatry. 2016 Jan 28;7:9. doi: 10.3389/fpsyt.2016.00009 (PMC4729935; doi:10.3389/fpsyt.2016.00009)
Supplement: Supplementary file 1 [file Presentation_1.PDF]

Supplementary Material to:

Revisiting the Basic Symptom Concept: Towards Translating Risk Symptoms for Psychosis into Neurobiological Targets.

Frauke Schultze-Lutter*, Martin Debbané, Anastasia Theodoridou, Stephen J. Wood, Andrea Raballo, Chantal Michel, Stefanie J. Schmidt, Jochen Kindler, Stephan Ruhrmann, Peter J. Uhlhaas

***Correspondence**: Frauke Schultze-Lutter: frauke.schultze-lutter@kjp.unibe.ch

Content:

- Supplementary Information 1 incl. Supplementary Figure 1: The basic symptom concept
- Supplementary Information 2 incl. Supplementary Figures 2 and 3: The role of basic symptoms in the development of the self-disorder concept
- Supplementary Information 3 incl. Supplementary Table 1 and Supplementary Figures 4, 5 and 6: The basic symptom dimensions in children and adolescents, and adults
- References included in Supplementary Material

Supplementary Information 1:
The basic symptom concept

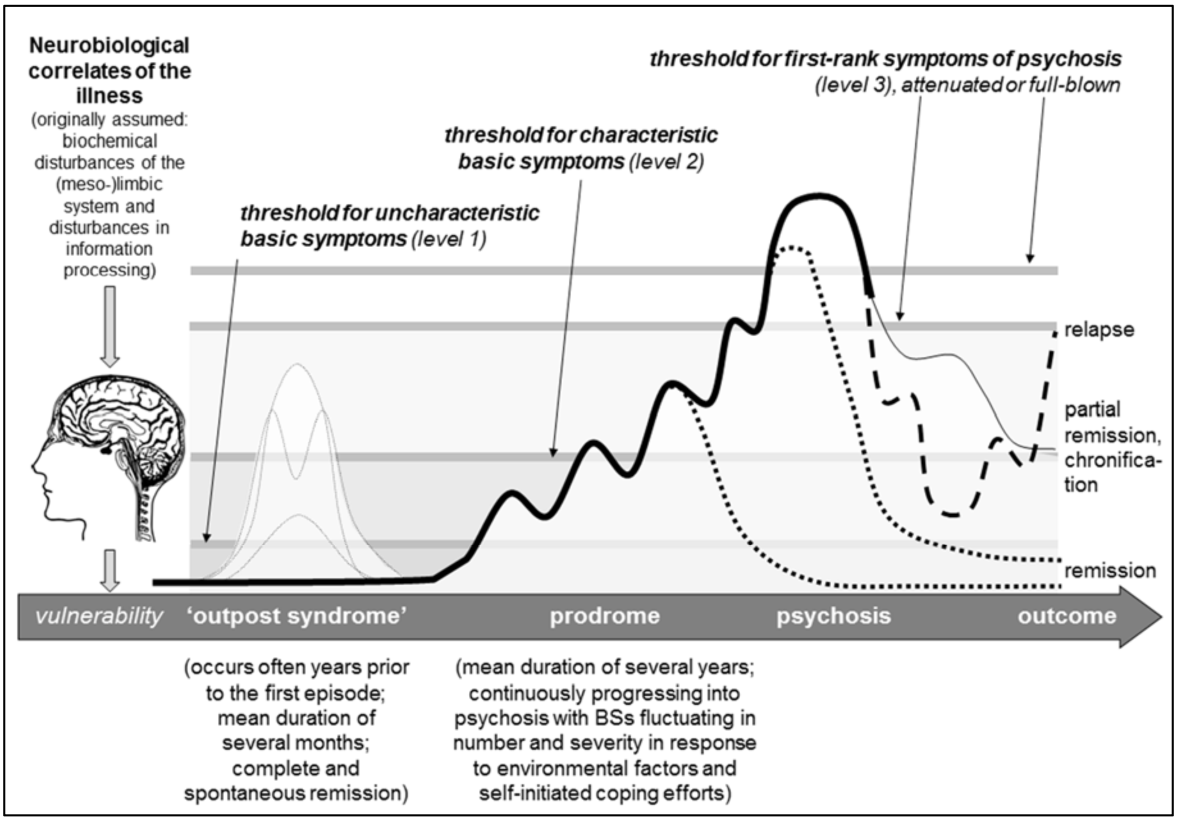

sFigure 1: The basic symptom (BSs) concept [acc. to Schultze-Lutter (1)]

Originally the basic symptoms (BSs) concept distinguished three permeable symptom levels of different specificity to psychoses (sFigure 1): uncharacteristic level 1 BSs affecting mainly drive, volition, affect, concentration and memory; characteristic, qualitatively peculiar level 2 BSs, especially of thinking, speech, (body) perception and motor action; and, at level 3, (attenuated) psychotic symptoms (2,3). The ability to experience BSs with insight and to report them often attenuates with progressive illness and emerging psychotic symptoms, but is restored upon remission (2).

The original BS concept (2,3) assumes that, upon debut at level 1, BSs will gradually increase in number and severity and, in most cases, will ultimately develop into psychotic symptoms, although temporary improvements are possible (thick line). In some cases, BSs will even completely remit spontaneously before reaching the threshold for full-blown psychotic symptoms (thin dotted lines on the left). These symptomatic phases that can mimic true prodromal stages announce the subsequent prodrome and, therefore, were called *outpost syndromes* (3).

Everyday situations and demands that overstrain an already pathologically vulnerable information processing capacity can trigger the development of characteristic level 2 BSs and their conversion to (attenuated) psychotic symptoms (2,3). Yet, if environmental and personal conditions are favourable (e.g., if there is a supportive social network, and the person possesses good social, problem solving and coping skills), BSs can be compensated for at any state almost completely as long as their number and/or severity do not overstrain personal resources (first thick dotted line). Thus in early phases, the emerging disorder will only become apparent to others when inadequate coping strategies are employed (e.g., social withdrawal or avoidance of certain situations/activities), when compensatory abilities are

exhausted and BSs start to interfere with behaviour as functional deficits and/or disorganizations of communication, or when insight cannot be maintained and, e.g., inadequate explanatory models are developed.
Following the first frank psychotic episode, BSs will evolve into one of three different types of outcome or *post-symptomatic basic stages* (on the right of the figure): (1) a reversible stage characterized by complete remission of BSs (thick dotted lines); (2) an irreversible symptomatic stage or *pure defect syndrome* with BSs, especially disturbances in drive, stress tolerance, affect and cognition, persisting on a level interfering with functioning (thin solid line); or (3) a prodromal stage of relapse developing from a low-symptom or even an asymptomatic state into a second episode (thick dashed line).

Supplementary Information 2: The role of basic symptoms in the development of the self-disorder concept

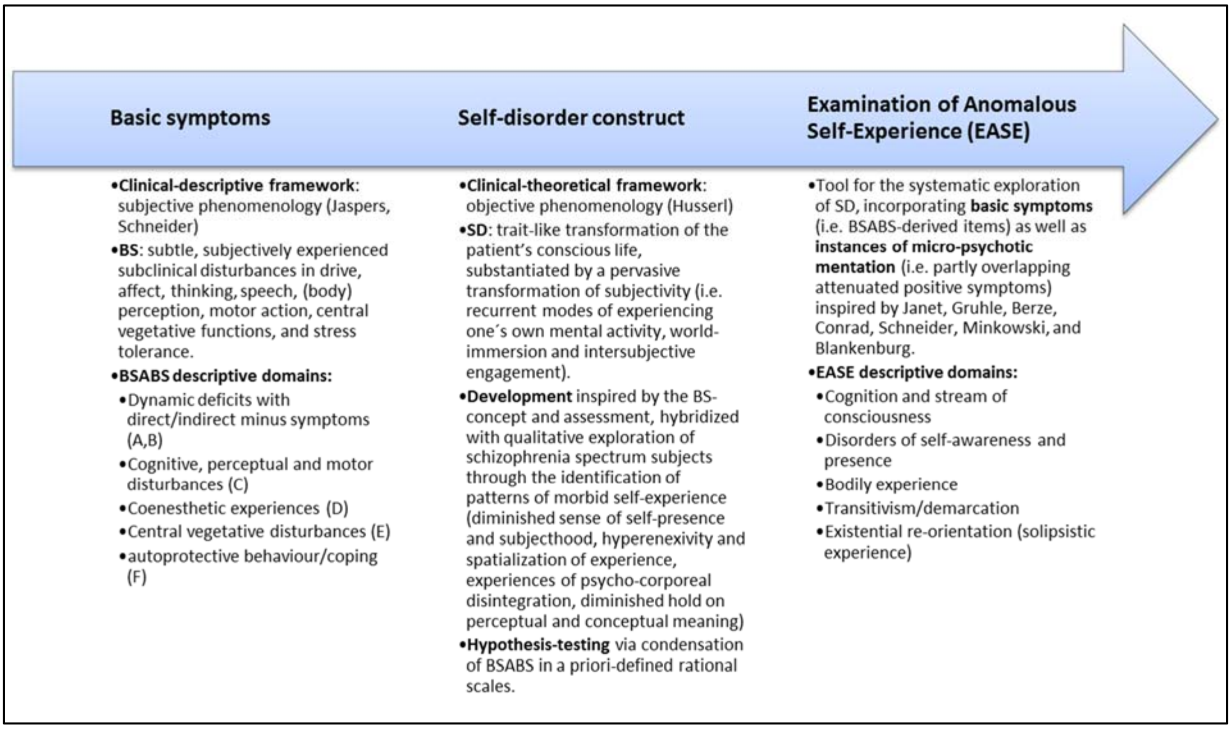

sFigure 2: **The development of the Examination of Anomalous Self-Experience** [EASE, (4)] **from the Bonn Scale for the Assessment of Basic Symptoms** [BSABS; (5)]

Inspired by the early research on BSs within the Copenhagen genetic high-risk study (6,7), clinical observations and philosophical considerations, the concept of "self-disorders" (SDs) was developed as a psychopathological construct in the 1990s, [see sFigure 2; (8)]. SDs are conceptualized as a constellation of interrelated anomalies of subjective experience gravitating around pervasive distortions of the "minimal" or "core self". The minimal self is a common concept in contemporary cognitive sciences, phenomenology and philosophy of mind that refers to the primary sense of "mineness", "myness" and "for-me-ness". This primary sense implicitly permeates the stream of experience and constitutes the primitive infrastructure or *medium* of the psychic life. Despite variable sensitivity and specificity of single EASE items for psychotic disorders (9-11), SDs were shown to agglomerate as a rather specific index of vulnerability to schizophrenia spectrum disorders (independently of the concomitant presence of overt psychotic symptoms) and also occur in schizotypal and schizotaxic conditions (12). After the initial validation of the SDs construct (conducted

through proxy-scores extrapolated from the BSABS) a specific assessment tool, the Examination of Anomalous Self-Experience [EASE; (4)], was developed on mainly theoretical grounds (sFigure 2). Despite some natural item overlaps with the BSABS [although even for analog items, definitions are usually slightly broader in the EASE – see Parnas et al. (4)], the EASE mainly focuses on *gestalt* transformations of the normal articulation of the stream of consciousness and incorporates also micro-psychotic experiential variants that phenomenologically overlap with APS as assessed by the Structured Interview for Psychosis-Risk Syndromes [SIPS; (13)] and the Comprehensive Assessment of At-Risk Mental States [CAARMS; (14)] (sFigure3).

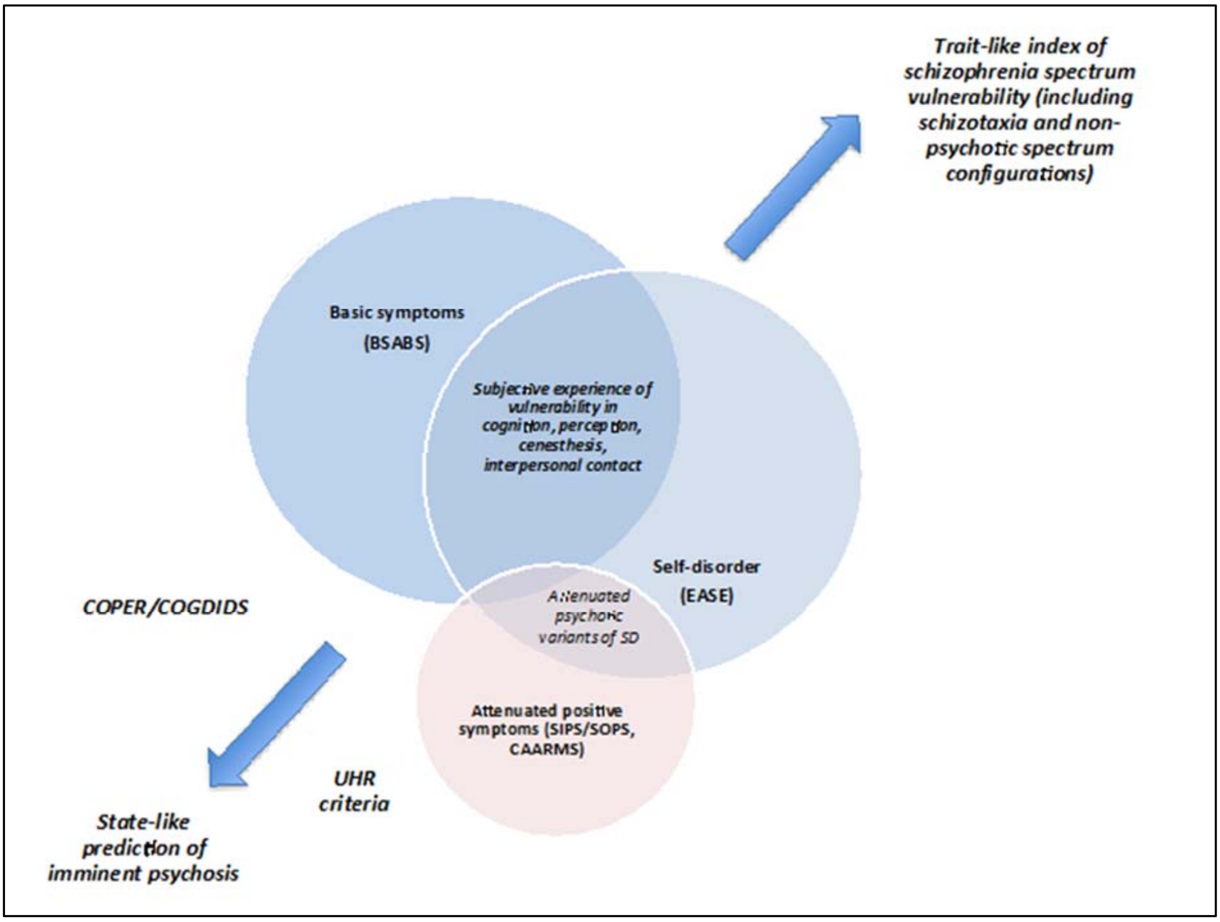

sFigure 3: Psychopathological overlap between BSs, SDs and APS and their respective assessments (BSABS, and more recently SPI-A/SPI-CY, EASE, SIPS and CAARMS)

Both state-like BSs as well as supposedly trait-like SDs manifest aspects of disturbed subjective experience that may (or may not) progress into "surface-level" psychotic symptoms. However, whereas the BSs concept and the related assessment tools were progressively refined for the prediction of imminent risk of psychosis, SDs were conceived as a core phenotype for the construct validity of the broad schizophrenia spectrum (in its clinical and non-clinical counterpart, e.g. schizotaxia and schizotypy).

Supplementary Information 3:
The basic symptom dimensions in children and adolescents, and adults

sTable 1: Comparison of BSs included in SPI-CY and SPI-A dimensions

**Children and adolescents: SPI-CY**	**Adults: SPI-A**
**Adynamia (A)**	**Mainly Affective-Dynamic Disturbances (A; all) and Cognitive-Attentional Impediments (B, 3 of 6)**
A1 Reduced energy and vitality (A.3.1.)	
A2 Reduced persistence, patience (A.3.2.)	
A3 Reduced drive and initiative (A.4.)	
A4 Impaired tolerance to certain stressors	A1 Impaired tolerance to certain stressors
A4.1. ... physical and/or mental labor (A.1./B1.1)	
A4.2 ... unusual, unexpected or specific novel demands (A.8.1./B.1.2.)	A1.1 … unusual, unexpected or specific novel demands (A.8.1./B.1.2.)
A4.3 ... certain social everyday situations (A.8.2./B.1.3.)	A1.2 … certain social everyday situations (A.8.2./B.1.3.)
A4.4 ... working under pressure of time or rapidly changing different demands (A.8.3./B.1.4.)	A1.3 … working under pressure of time or rapidly changing different demands (A.8.3./B.1.4.)
A5 Change in mood and emotional responsiveness (A.6.1.)	A2 Change in mood, emotional responsiveness (A.6.1.)
A6 Decrease in positive emotional responsiveness towards others (A.6.3.)	A3 Decrease in positive emotional responsiveness towards others (A.6.3.)
A7 Intermittent, recurrent depressive mood swings (A.6.5.)	
A8 Disturbance in presenting oneself (A.7.2.)	
A9 Increased emotional reactivity in response to everyday events (B.2.1.)	
A10 Increased emotional reactivity in response to routine social interactions that affect the young person directly or indirectly (B.2.2.)	D2 Increased emotional reactivity in response to routine social interactions (B.2.2.)
A11 Difficulties concentrating (C.1.5.)	B3 Difficulties concentrating (C.1.5.)
A12 Forgetfulness, scatterbrainedness (C1.11.)	
A13 Slowed-down thinking (C.1.12.)	B5 Slowed-down thinking (C.1.12.)
A14 Lack of 'thought energy' or goal-directed thoughts (from age 13 onwards) (C.1.13.)	B6 Lack of 'thought energy', purposive thoughts (C.1.13.)
**Perception Disturbances (B)**	**Mainly Body Perception Disturbances (E; 4 of 6), Perception Disturbances (F; 3 of 6) and Optional items (O; 13 of 21)**
B1 Decreased ability to discriminate between ideas and perception, fantasy and true memories (C.1.15.)	O2 Decreased ability to discriminate between ideas and perception, fantasy and true memories (C.1.15.)
B2 Unstable ideas of reference (C.1.17.)	D4 Unstable ideas of reference (C.1.17.)
B3 Visual perception disturbances	O4 Other visual perception disturbances

**Children and adolescents: SPI-CY**	**Adults: SPI-A**
B3.1 Near and tele-vision (C.2.3.1.)	O4.1 Near and tele-vision (C.2.3.1.)
B3.2 Micropsia, macropsia (C.2.3.2.)	F3 Micropsia, macropsia (C.2.3.2.)
B3.3 Metamorphopsia (C.2.3.3.)	O4.2 Metamorphopsia (C.2.3.3.)
B3.4 Changes in color vision (C.2.3.4.)	O4.3 Changes in color vision (C.2.3.4.)
B3.5 Changed perception of the face or body of others (C.2.3.5.)	D5 Changed perception of the face or body of others (C.2.3.5.)
B3.6 Changed perception of the own face (C.2.3.6.)	O4.4 Changed perception of patient's own face (C.2.3.6.)
B3.7 Pseudomovements of optic stimuli (C.2.3.7.)	O4.5 Pseudomovements of optic stimuli (C.2.3.7.)
B3.8 Diplopsia, oblique vision (C.2.3.8.)	O4.6 Diplopsia, oblique vision (C.2.3.8.)
B3.9 Disturbances of the estimation of distances or sizes (C.2.3.9.)	O4.7 Disturbances of the estimation of distances or sizes (C.2.3.9.)
B3.10 Disturbances of the perception of straight lines or contours (C.2.3.10.)	O4.8 Disturbances of the perception of straight lines/contours (C.2.3.10.)
B3.11 Dysmegalopsia (C.2.3.11.)	
B3.12 Maintenance of visual stimuli, ‚visual echoes' (C.2.3.12.)	O4.9 Maintenance of optic stimuli, 'visual echoes' (C.2.3.12.)
B4 Hypersensitivity to sounds and acoasms	Acoustic hypersensitivity and acoasms
B4.1 Hypersensitivity to sounds or noise (C.2.4.1.)	F4 Hypersensitivity to sounds / noise (C.2.4.1.)
B4.2 Acoasms (C.2.4.2.)	O5.1 Acoasms (C.2.4.2.)
B5 Other acoustic perception disturbances	Other acoustic perception disturbances
B5.1 Changes in perceived intensity or quality of acoustic stimuli (C.2.5.1.)	F5 Changed intensity/quality of acoustic stimuli (C.2.5.1.)
B5.2 Maintenance of acoustic stimuli, ‚acoustic echoes' (C.2.5.2.)	O5.2 Maintenance of acoustic stimuli, 'acoustic echoes' (C.2.5.2.)
B6 Disturbance of the comprehension of visual or acoustic stimuli (C.2.7.)	
B7 Derealization (from age 13 onwards) (C.2.11.)	O8 Derealization (C.2.11.)
B8 Body perception disturbances	E Body Perception Disturbances
B8.1 Unusual bodily sensations of numbness and stiffness (D.1.)	E1 Bodily sensations of numbness and stiffness (D.1.)
B8.2 Somatopsychic bodily depersonalization (D.1.1.)	F6 Somatopsychic bodily depersonalization (D.1.1.)
B8.3 Migrating bodily sensations wandering through the body (D.4.)	E3 Bodily sensations migrating through the body (D.4.)
B8.4 Electric bodily sensations, feelings of being electrified (D.5.)	E4 Bodily sensations of being electrified (D.5.)
B8.5 Bodily sensations of movement, pulling or pressure inside the body or on its surface (D.7.)	E5 Bodily sensations of movement or pressure (D.7.)
B8.6 Bodily sensations of abnormal heaviness, lightness, emptiness, falling, sinking, levitation or elevation (D.8.)	

Children and adolescents: SPI-CY	Adults: SPI-A
	E6 Sensations of the body or parts of it extending, diminishing, shrinking, enlarging, growing, or constricting (D.9.)
**Neuroticism (C)**	**No counterpart**
C1 Decreased need for social contacts (A.6.4.)	
C2 Increased emotional reactivity in response to adversities of strangers (B.2.3.)	
C3 Increased excitability and irritability (B.2.4.)	
C4 Obsessive-compulsive phenomena (B3.2.)	
C5 Phobic phenomena (B.3.3.)	
C6 Autopsychic depersonalization (from age 13 onwards) (B3.4.)	
C7 Unusual bodily sensations of pain in a distinct area (D.3.)	E2 Bodily sensations of pain in a distinct area (D.3.)
C8 Dysesthesias caused by touch or perceptions (D.12.)	
**Thought and Motor Disturbances (D)**	**Mainly Cognitive Disturbances (C; all), Cognitive-Attentional Impediments (B; 3 of 6) and Optional items (O; 5 of 21)**
D1 Increased indecisiveness with regard to insignificant choices between equal alternatives (from age 13 onwards). (A.5.)	C1 Increased indecisiveness with regard to insignificant choices between equal alternatives (A.5.)
D2 Impaired social skills (A.7.1.)	
D3 Decreased spontaneity, increased self-reflection (from age 13 onwards) (B.3.1.)	
D4 Disturbances of immediate recall (C.1.8.)	C6 Disturbance of immediate recall (C.1.8.)
D5 Difficulties to hold things in mind for less than an hour (C.1.9.)	B4 Difficulties to hold things in mind for less than half an hour (C.1.9.)
D6 Disturbance in retrieving knowledge from long-term memory (from age 13 onwards) (C.1.10.)	
D7 Disturbances of abstract thinking (from age 13 onwards) (C.1.16.)	O3 Disturbances of abstract thinking (C.1.16.)
D8 Inability to divide attention (A.8.4.)	B1 Inability to divide attention (A.8.4.)
D9 Thought interference (C.1.1.)	C2 Thought interference (C.1.1.)
D10 Thought pressure (C.1.3.)	D3 Thought pressure (C.1.3.)
D11 Disturbance of receptive speech (C.1.6.)	C4 Disturbance of receptive speech (C.1.6.)
D12 Disturbances of expressive speech (C.1.7.)	C5 Disturbance of expressive speech (C.1.7.)
D13 Decreased capacity to discriminate between different kinds of emotions (A.6.2.)	D1 Decreased capacity to discriminate between different kinds of emotions (A.6.2.)
D14 Thought perseveration (C.1.2.)	O1 Thought perseveration (C.1.2.)
D15 Thought blockages (from age 13 onwards) (C.1.4.)	C3 Thought blockages (C.1.4.)

**Children and adolescents: SPI-CY**	**Adults: SPI-A**
D16 Feeling overly distracted by stimuli (C.2.8.)	B2 Feeling overly distracted by stimuli (C.2.8.)
D17 Motor interference exceeding simple lack of co-ordination (C.3.1.)	O9 Motor interference exceeding simple lack of co-ordination (C.3.1.)
D18 Motor blockages (C.3.2.)	O10 Motor blockages (C.3.2.)
D19 Loss of automatic skills (C.3.3.)	O11 Loss of automatic skills (C.3.3.)
**Optional Items (O) part of BS criteria**	**Optional Items (O) with a positive predictive value of equal or greater 0.70 according to the CER study**
O1 Partial seeing including tubular vision (C.2.1.3.)	O4.10 Partial seeing incl. tubular vision (C.2.1.3.)
O2 Captivation of attention by details of the visual field (C.2.9.)	O7 Captivation of attention by details of the visual field (C.2.9.)
O3 Photopsia (C.2.2.2.)	F2 Photopsia (C.2.2.2.)
	F1 Hypersensitivity to light or certain optic stimuli (C.2.2.1.)

Numbers in parentheses refer to the BSABS (5) which was the source instrument for the Schizophrenia Proneness Instrument, Adult [SPI-A; (15)] and Child and Youth version [SPI-CY;(16)] as well as the main source instrument for the EASE.
BSs included in COPER and/or COGDIS are highlighted in yellow.

The SPI-A was generated and validated based on longitudinal and cross-sectional BSABS data of 79 truly prodromal adult patients of the Cologne Early Recognition, CER, study (17) using cluster and Facetted Smallest Space analyses (FSSA) (18). In Procrustean Individual Differences Scaling analyses, the generated six BS dimensions in adult samples proofed a rather robust structure across different stages of the illness, i.e., between prodromal and frankly psychotic, yet non-chronic states. This structure even remained largely unchanged when BS assessment turned from a binary assessment of presence (as done in the BSABS) to an ordinal assessment of severity (as done in the SPI-A and SPI-CY), yet, it could not be replicated in a sample of patients with non-psychotic, depressive disorders (18). Thus, it was concluded that these dimensions might be inherent to schizophrenia across different stages of the illness and might offer a good starting point for the development of an instrument for the assessment of prodromal symptoms that occur early in course of the illness, thereby serving as valid and reliable subscales (18). Taking on from this, the final version of the SPI-A with six subscales was developed after validation on a second prospectively assessed truly prodromal sample of 51 patients meeting COPER at baseline and subsequently developing psychosis within 2 years (sFigure 4).

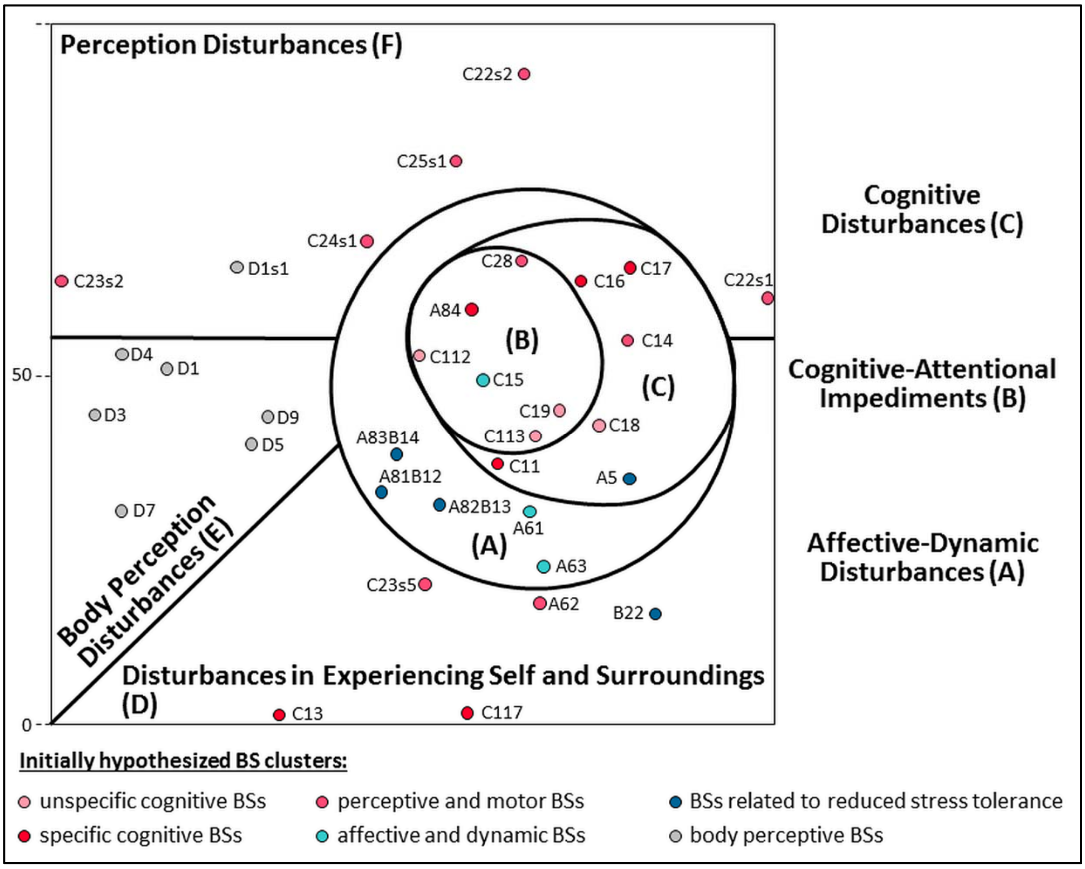

sFigure 4: Construct validation of SPI-A dimensions.
Axional result of confirmatory FSSA of a truly prodromal group (N=51); good separation index of 0.85.

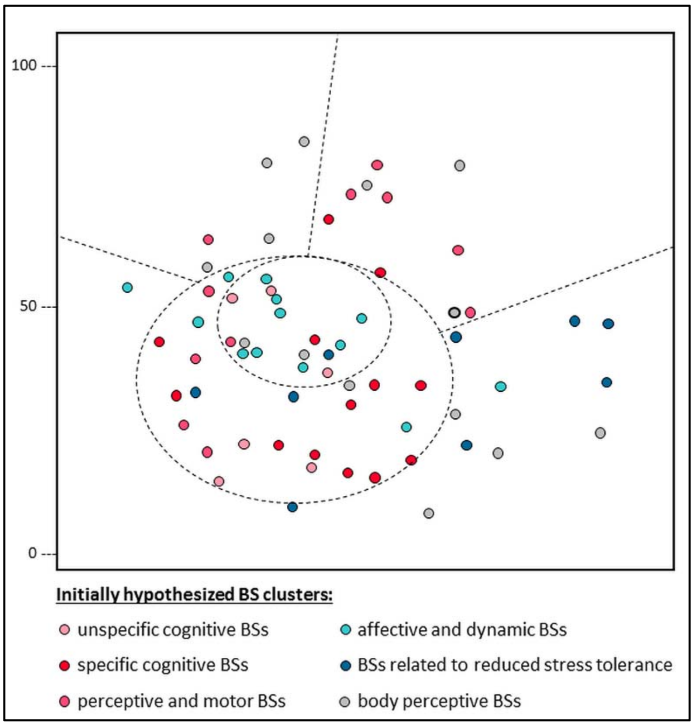

Though amazingly similar across adult samples and even robust against the change from the BSABS's binary rating of presence to the SPI's ordinal rating of severity, this structure could not be replicated in an early-onset psychosis sample with an onset of psychosis before the age of 18 (19) (sFigure 5).

sFigure 5, left: **Result of confirmatory FSSA of an early-onset psychosis group** (N=32); poor separation index of 0.25 as indicated by the lack of clustering of hypothesized BS clusters.

The dimensional space of BSs fitting the data of the early-onset psychosis sample best is displayed in sFigure 6 and was used to define the subscales of the SPI-CY (sTable 1).

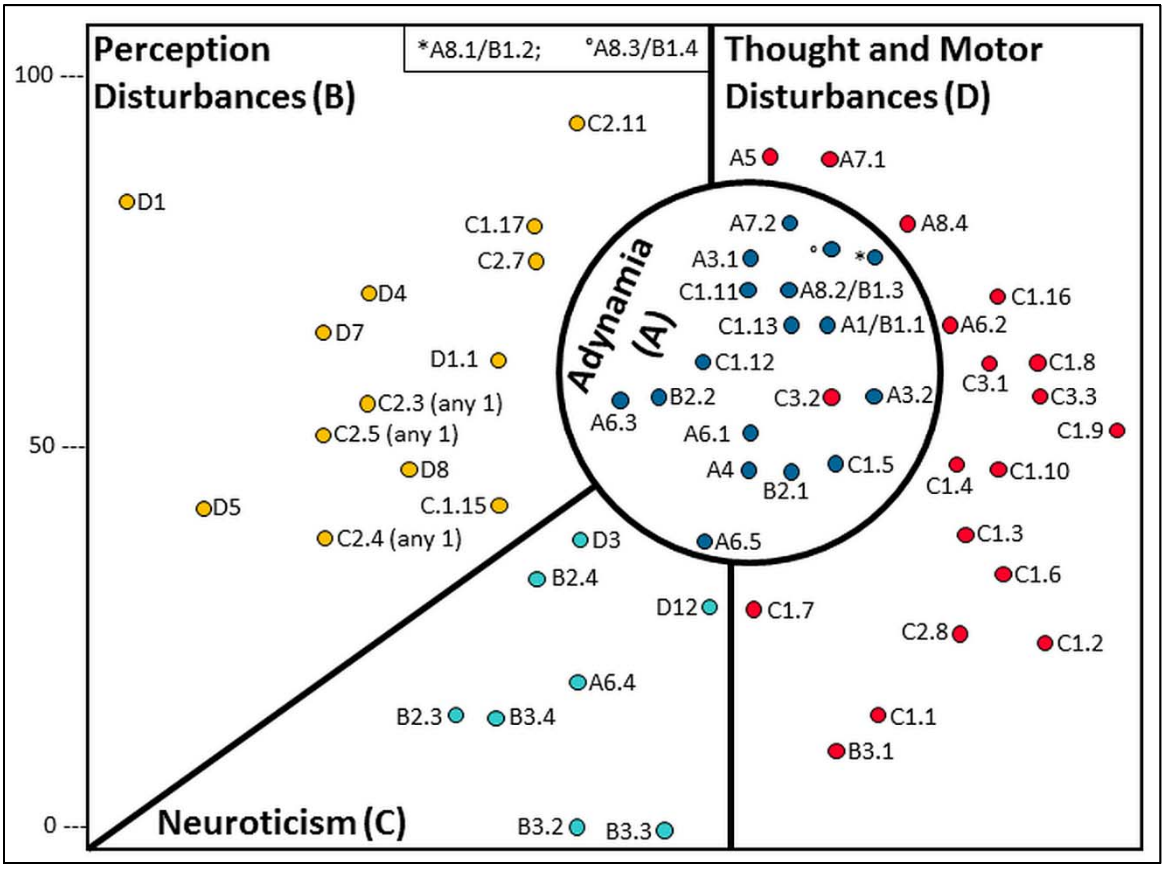

sFigure 6: **Result of the revised confirmatory FSSA of an early-onset psychosis group** (N=32); excellent separation index of 0.93.

Despite their differences across ages or developmental states, the solutions had some important features in common: (1) they distinguished between more psychosis-specific cognitive BSs that are part of COPER and COGDIS (Table 1) and rather unspecific cognitive BSs, such as memory or concentration difficulties, hyperdistractibility and decrease in thought energy or initiation; (2) unspecific cognitive BSs were proximate to adynamic-affective BSs, such as decrease of energy, drive, initiative, interest or patience, impaired stress tolerance and affective disturbances; and (3) sensory and body perception disturbances were closely linked (20,21).

References included in Supplementary Information

1. Schultze-Lutter F. Subjective symptoms of schizophrenia in research and the clinic: the basic symptom concept. *Schizophr Bull* (2009) **35**(1):5-8. doi:10.1093/schbul/sbn139.
2. Gross, G. The "basic" symptoms of schizophrenia. *Br J Psychiatry* (1989) **155**(Suppl 7):21-5.
3. Huber G, Gross G. The concept of basic symptoms in schizophrenic and schizoaffective psychoses. *Recenti Prog Med* (1989) **80**:646-52.
4. Parnas J, Møller P, Kircher T, Thalbitzer J, Jansson L, Handest P, Zahavi D. EASE: Examination of Anomalous Self-Experience. *Psychopathology* (2005) **38**(5):236-58. doi:10.1016/S0013-7006(12)70090-8
5. Gross G, Huber G, Klosterkötter J. *Bonner Skala für die Beurteilung von Basissymptomen (BSABS; Bonn Scale for the Assessment of Basic Symptoms)* Berlin: Springer (1987). 199 p.
6. Parnas J. From predisposition to psychosis: progression of symptoms in schizophrenia. Acta Psychiatr Scand (1999) 99(suppl. 395):20-9.
7. Parnas J, Mednick SA. "Early predictors of onset and course of schizophrenia and schizophrenia spectrum". In: Häfner H, Gattaz WF, editors. *Search for the causes of schizophrenia, Vol 2*. Berlin Heidelberg New York, Springer: (1991). p. 34-47.
8. Parnas J, Jansson L, Sass LA, Handest P. Self-experience in the prodromal phases of schizophrenia: a pilot study of first admissions. *Neurol Psychiatry Brain Res* (1998) **6**:107–16.
9. Haug E, Lien L, Raballo A, Bratlien U, Oie M, Andreassen OA, et al. Selective aggregation of self-disorders in first-treatment DSM-IV schizophrenia spectrum disorders. *J Nerv Ment Dis* (2012) **200**(7):632-6. doi:10.1097/NMD.0b013e31825bfd6f
10. Nordgaard J, Parnas J. Self-disorders and the schizophrenia spectrum: a study of 100 first hospital admissions. *Schizophr Bull* (2014) **40**(6):1300-7. doi:10.1093/schbul/sbt239
11. Møller P, Haug E, Raballo A, Parnas J, Melle I. Examination of anomalous self-experience in first-episode psychosis: interrater reliability. *Psychopathology* (2011) **44**(6):386-90. doi:10.1159/000325173
12. Raballo A, Parnas J. The Silent Side of the Spectrum: Schizotypy and the Schizotaxic Self. *Schizophr Bull* (2011) **37**(5):1017-26. doi:10.1093/schbul/sbq008
13. McGlashan T, Walsh B, Woods S. *The psychosis-risk syndrome. Handbook for diagnosis and follow-up*. New York, NY: Oxford University Press (2010). 256p.
14. Yung AR, Yuen HP, McGorry PD, Phillips LJ, Kelly D, Dell'Olio M, et al. Mapping
15. the onset of psychosis: the comprehensive assessment of at-risk mental states. *Aust N Z J Psychiatry* (2005) **39**(11–12):964–71. doi:10.1080/j.1440-1614.2005.01714.x
16. Schultze-Lutter F, Addington J, Ruhrmann S, Klosterkötter J. *Schizophrenia Proneness Instrument, Adult version (SPI-A).* Rome: Giovanni Fioriti Editore s.r.l. (2007). 97 p.
17. Schultze-Lutter F, Koch E. *Schizophrenia Proneness Instrument, Child & Youth version (SPI-CY).* Rome: Giovanni Fioriti Editore s.r.l. (2010). 104 p.
18. Klosterkötter J, Hellmich M, Steinmeyer EM, Schultze-Lutter F. Diagnosing schizophrenia in the initial prodromal phase. *Arch Gen Psychiatry* (2001) **58**(2):158-64. doi:10.1001/archpsyc.58.2.158
19. Schultze-Lutter F, Steinmeyer E, Ruhrmann S, Klosterkötter J. The dimensional structure of self-reported prodromal disturbances in schizophrenia. *Clin Neuropsychiatry* (2008) **5**(3):140-50.

20. Koch E, Schultze-Lutter F, Schimmelmann BG, Resch F. On the importance and detection of prodromal symptoms from perspective of child and adolescent psychiatry. *Clin Neuropsychiatry* (2010) **7**(2):38-48.
21. Schultze-Lutter F, Steinmeyer EM, Ruhrmann S, Klosterkötter J. The dimensional structure of self-reported ‘prodromal’ disturbances in schizophrenia. *Clin Neuropsychiatry* (2008) **5**:140-50.
22. Schultze-Lutter F, Ruhrmann S, Fusar-Poli P, Bechdolf A, Schimmelmann BG, Klosterkötter J. Basic symptoms and the prediction of first-episode psychosis. *Curr Pharm Des* (2012) **18**(4):351-7.
